# Astrocytosis precedes amyloid plaque deposition in Alzheimer APPswe transgenic mouse brain: a correlative positron emission tomography and in vitro imaging study

**DOI:** 10.1007/s00259-015-3047-0

**Published:** 2015-04-17

**Authors:** Elena Rodriguez-Vieitez, Ruiqing Ni, Balázs Gulyás, Miklós Tóth, Jenny Häggkvist, Christer Halldin, Larysa Voytenko, Amelia Marutle, Agneta Nordberg

**Affiliations:** Division of Translational Alzheimer Neurobiology, Centre for Alzheimer Research, Department of Neurobiology, Care Sciences and Society, Karolinska Institutet, Novum 5th Floor, Blickagången 6, 141 57 Stockholm, Sweden; Centre for Psychiatric Research, Department of Clinical Neuroscience, Karolinska Institutet, Stockholm, Sweden; NTU – Imperial College, Lee Kong Chian School of Medicine, Nanyang Technological University, Singapore, Singapore; Department of Geriatric Medicine, Karolinska University Hospital Huddinge, Stockholm, Sweden

**Keywords:** Alzheimer’s disease, APPswe mice, Amyloid, Astrocytosis, ^11^C-AZD2184, ^11^C-Deuterium-L-deprenyl, PET imaging

## Abstract

**Purpose:**

Pathological studies suggest that neuroinflammation is exacerbated by increased beta-amyloid (Aβ) levels in the brain early in Alzheimer’s disease (AD). The time course and relationships between astrocytosis and Aβ deposition were examined using multitracer in vivo positron emission tomography (PET) imaging in an AD transgenic mouse model, followed by postmortem autoradiography and immunohistochemistry analysis.

**Methods:**

PET imaging with the amyloid plaque tracer ^11^C-AZD2184 and the astroglial tracer ^11^C-deuterium-L-deprenyl (^11^C-DED) was carried out in APPswe mice aged 6, 8–15 and 18–24 months (4–6 animals/group) and in wild-type (wt) mice aged 8–15 and 18–24 months (3–6 animals/group). Tracer uptake was quantified by region of interest analysis using PMOD software and a 3-D digital mouse brain atlas. Postmortem brain tissues from the same APPswe and wt mice in all age groups were analysed for Aβ deposition and astrocytosis by in vitro autoradiography using ^3^H-AZD2184, ^3^H-Pittsburgh compound B (PIB) and ^3^H-L-deprenyl and immunostaining performed with antibodies for Aβ_42_ and glial fibrillary acidic protein (GFAP) in sagittal brain sections.

**Results:**

^11^C-AZD2184 PET retention in the cerebral cortices of APPswe mice was significantly higher at 18–24 months than in age-matched wt mice. Cortical and hippocampal ^11^C-DED PET binding was significantly higher at 6 months than at 8–15 months or 18–24 months in APPswe mice, and it was also higher than at 8–15 months in wt mice. In vitro autoradiography ^3^H-AZD2184 and ^3^H-PIB binding confirmed the in vivo findings with ^11^C-AZD2184 and demonstrated age-dependent increases in Aβ deposition in APPswe cortex and hippocampus. There were no significant differences between APPswe and wt mice in ^3^H-L-deprenyl autoradiography binding across age groups. Immunohistochemical quantification demonstrated more Aβ_42_ deposits in the cortex and hippocampus and more GFAP^+^ reactive astrocytes in the hippocampus at 18–24 months than at 6 months in APPswe mice.

**Conclusion:**

The findings provide further in vivo evidence that astrocytosis occurs early in AD, preceding Aβ plaque deposition.

**Electronic supplementary material:**

The online version of this article (doi:10.1007/s00259-015-3047-0) contains supplementary material, which is available to authorized users.

## Introduction

Alzheimer’s disease (AD) is the most common neurodegenerative disease and dementia disorder. Beta-amyloid (Aβ) plaques, neurofibrillary tangles, neuroinflammation and neuronal loss in the brain are pathological hallmarks of AD that are thought to play a key role in the progressive decline in episodic memory and cognition [1, 2]. Recent advancements in molecular imaging using positron emission tomography (PET) have allowed the visualization of fibrillar Aβ plaques and the monitoring of disease progression in vivo in AD patients [3]. Amyloid tracers developed for human use include ^11^C-Pittsburgh compound B (^11^C-PIB) [4], ^11^C-BF-227 [5], ^11^C-AZD2184 [6], ^18^F-FDDNP [7], ^18^F-florbetapir [8], ^18^F-flutemetamol [9], ^18^F-florbetaben [10] and ^18^F-AZD4694 [11]. Recently, ^18^F-florbetapir, ^18^F-flutemetamol and ^18^F-florbetaben have been approved by the European Medicines Agency (EMA) and by the US Food and Drug Administration (FDA) to visualize amyloid plaques. We recently reported that florbetaben, PIB and florbetapir all bind to a high-affinity binding site in postmortem AD brain tissues [12], demonstrating their reliability for detecting fibrillar Aβ deposits. Longitudinal amyloid PET imaging in large at-risk and patient populations has shown that it takes ≈ 20 years for build-up of pathological amyloid plaque load in the brain [13].

Neuroinflammation is increasingly recognized to play an early role in AD [14–16]. Studies in postmortem AD brains have demonstrated abundant reactive astrocytes and microglia around amyloid plaques [17], but little is known about their in vivo distribution or function. PET imaging of astrocytosis in subjects with mild cognitive impairment (MCI) and in AD patients using ^11^C-deuterium-L-deprenyl (^11^C-DED) has suggested that astrocytosis occurs early in AD [18].

A wide range of transgenic mice harbouring familial AD mutations that model different aspects of AD pathogenesis are currently available. Although none of these fully replicates the disease, they have provided important insights into the pathophysiology of Aβ toxicity. The development of pathology varies among the mouse strains. APPswe mice carrying the APP Swedish mutation develop amyloid pathology more slowly than APP/PS1 and APP23 mice [19].

In this study, we investigated the time course of astrocytosis and amyloid plaque deposition by multitracer in vivo microPET imaging in APPswe mice aged 6–24 months. The postmortem brains were then analysed using correlative immunohistochemistry and autoradiography.

## Materials and methods

### Animals

Male and female transgenic mice expressing the APP Swedish mutation (Tg2576) were obtained by in-house breeding at the Karolinska Institutet animal care facility, as previously described [20]. Wild-type (wt) littermates served as controls. All mice were housed under the same conditions with access to food and water ad libitum and a 12-h light/dark cycle. All experimental procedures complied with the guidelines and regulations of the Swedish National Board for Laboratory Animals, and the Regional Ethics and Animal Research Committee at Karolinska Institutet approved the study protocol.

### PET imaging in APPswe and wt mice

A nanoScan® small animal PET/magnetic resonance imaging (PET/MRI) and a PET/computed tomography (PET/CT) scanner system (Mediso Ltd, Budapest, Hungary) [21, 22] were used to image the brains of 6-, 8- to 15- and 18- to 24-month-old APPswe mice (*n* = 4–6 animals in each age group) and 8- to 15- and 18- to 24-month-old wt mice (*n* = 3–6 animals in each age group) at the Karolinska Experimental Research and Imaging Centre (KERIC). The PET radiotracers ^11^C-AZD2184 and ^11^C-DED were synthesized at Karolinska PET Radiochemistry Laboratory in a similar way as reported elsewhere [23, 24], with a radiochemical purity >98 % and specific radioactivity of 181 ± 211 and 373 ± 443 (mean ± SD) GBq/μmol, respectively. The mice were anaesthetized with 1.5 % (v/v) isoflurane and each mouse underwent an MRI or CT scan for anatomical reference. ^11^C-AZD2184 (10.6 ± 1.8 MBq) and ^11^C-DED (10.5 ± 1.6 MBq) were administered by venous tail injections and dynamic PET scans were acquired over 63 min in 3-D list mode. The injected mass was 0.020 ± 0.014 μg (^11^C-AZD2184) and 0.028 ± 0.037 μg (^11^C-DED); the corresponding injected quantities expressed in nanomolar units were 0.098 ± 0.051 nmol (^11^C-AZD2184) and 0.174 ± 0.242 nmol (^11^C-DED). Most mice had PET scans with both tracers, with a 6- to 15-day interval between scans.

#### PET image data reconstruction and processing

The PET data were corrected for decay and dead time. The image reconstruction protocol consisted of a 400–600 keV energy window with a 5 ns time window, using a 20-iteration maximum likelihood expectation maximization (MLEM) algorithm and a 1 × 10^−4^ regularization parameter. The PET data were reconstructed into 25 time frames: 4 × 10, 4 × 20, 4 × 60, 7 × 180 and 6 × 360 s, with an isovoxel of 0.3 mm. PMOD v3.3 software (PMOD Technologies Ltd, Zurich, Switzerland) was used for the coregistration of the PET images to a 3-D digital T2-weighted MRI mouse brain template incorporated in PMOD. To facilitate the coregistration of dynamic PET data, two separate averaged PET images, i.e. the integrated early (0–4 min) and late (4–63 min) frames, were obtained. The early frames were manually coregistered to the mouse brain template available in PMOD for each mouse PET image. The corresponding transformation matrix was subsequently applied to the averaged late-frames PET image, and the manual coregistration was further improved. The final transformation matrix was then applied to the whole dynamic PET sequence for each mouse, resulting in PET images that were coregistered to the mouse brain template in PMOD.

#### Quantification of PET tracer uptake

Coregistered PET images were analysed by region of interest (ROI) analysis using a 3-D mouse brain atlas in PMOD. An averaged 21- to 33-min frame was used for quantifying the extent of ^11^C-AZD2184 and ^11^C-DED radiotracer uptake, and the averaged activity in each target ROI was subsequently corrected for injected radioactivity and weight of the mice and expressed in standardized uptake value (SUV) units. The cortex, bilateral hippocampus and cerebellum were selected for further evaluation. Postmortem immunohistochemical analysis of APPswe mouse brains revealed the presence of relatively few diffuse plaques in the cerebellum, which also differed morphologically from those in the cortex and hippocampus. The cerebellum was thus not used as a reference for quantifying ^11^C-AZD2184 retention. Astrocytes were more sparse in the cerebellum than in the cortex and hippocampus, and ^11^C-DED binding was quantified using a Simplified Reference Tissue Model (SRTM) in PMOD using the cerebellum as a reference and expressed as non-displaceable binding potential (BP_ND_) for each ROI. In addition, ^11^C-DED was also expressed by its semi-quantitative target to cerebellum ratio in SUV ratio (SUVR) units for each ROI.

### In vitro homogenate binding and autoradiography in APPswe and wt mice postmortem brains

Within 2 weeks after undergoing one ^11^C-AZD2184 and one ^11^C-DED PET scan, each mouse was sacrificed by cervical dislocation and its brain removed for postmortem analyses. Right brain hemispheres were stored at –80 °C and used in homogenate binding and autoradiography assays. Left brain hemispheres were post-fixed with 4 % paraformaldehyde (pH 7.4), transferred to a sucrose cryoprotectant for 24 h at 4 °C and then frozen at –80 °C for immunohistochemistry.

Binding assays with ^3^H-AZD2184 (specific activity 21.9 Ci/mmol, custom synthesized at Karolinska PET Radiochemistry Laboratory, Stockholm, Sweden), ^3^H-PIB (specific activity 85 Ci/mmol) and ^3^H-L-deprenyl (specific activity 80 Ci/mmol, both custom synthesized by Quotient Bioresearch, Cardiff, UK) were performed on cortical homogenates (50–100 μg tissue) from APPswe mice. Autoradiography was performed by incubation of triplicate sagittal sections (10 μm) from APPswe and wt mouse brains with 3 nM ^3^H-AZD2184, 1.5 nM ^3^H-PIB and 10 nM ^3^H-L-deprenyl, according to methods described previously [25, 26]. Adjacent sections were incubated with unlabelled 1 μM BTA-1 or 10 μM L-deprenyl (Sigma-Aldrich, St. Louis, MO, USA) to determine non-specific binding. The autoradiograms were analysed with Multigauge software V3.0 (Fuji, Tokyo, Japan) and specific binding values were expressed as picomoles per gram tissue. Selected ROIs were the cerebral cortex, the hippocampus and the cerebellum.

### Immunohistochemistry

APPswe and wt mice brain sections were immunostained with mouse monoclonal Aβ_42_ (1:200; Signet Laboratories, Dedham, MA, USA), and polyclonal rabbit glial fibrillary acidic protein (GFAP) (1:500; DakoCytomation, Glostrup, Denmark) antibody. Controls consisted of brain sections treated with either non-immune serum or omission of the primary antibody. Sections were imaged sequentially at ×10 and ×20 magnification under light microscopy (Leica, Wetzlar, Germany) with an attached image capture analysis system (ProgRes Capture Pro 2.8.8 software, JenOptik AG, Jena, Germany). Quantification was performed using Image J software (National Institutes of Health, Bethesda, MD, USA) and the results expressed as the number of immunopositive cells per square millimetre.

### Statistics

Statistical analyses of the in vivo PET data, in vitro binding assays and postmortem immunohistochemistry analyses were performed using SPSS (IBM SPSS Statistics, Version 22.0) and GraphPad Prism version 5.0 software. Correlations between regional PET tracer uptake and age of mice were performed by non-parametric Spearman’s rank-order correlation method. Comparisons between the APPswe and wt mice according to age group were performed by independent sample, one- or two-tailed, non-parametric Mann-Whitney U tests. Results were presented as mean ± SD for each study group. Significant differences between groups were indicated by *p < 0.05, **p < 0.01 and ***p < 0.001.

## Results

The demographics of ^11^C-AZD2184 and ^11^C-DED in vivo PET-imaged APPswe and wt mice are shown in Table 1. Visual inspection of the PET time-activity curves (TACs) for each ROI showed that both tracers were relatively homogeneously distributed throughout the brain. The uptake of both tracers peaked rapidly at 30–40 s post-injection and they were washed out relatively quickly so that the level of radioactivity had dropped after about 4 min post-injection (Suppl. Fig. S1).

**Table 1:** Demographics of in vivo ^11^C-AZD2184 and ^11^C-DED microPET studies

Study groups		^11^C-AZD2184 PET				^11^C-DED PET			
Mouse strain	Age (months)	*n*	Sex (M/F)	Weight (g)	Inj. dose (MBq)	*n*	Sex (M/F)	Weight (g)	Inj. dose (MBq)
APPswe	6	4	4/0	24.8 ± 6.1	9.3 ± 2.0	4	4/0	25.0 ± 5.4	8.9 ± 1.5
APPswe	8–15	6	6/0	29.7 ± 3.3	10.4 ± 2.0	6	6/0	33.2 ± 4.9	11.1 ± 1.6
APPswe	18–24	5	3/2	23.0 ± 3.9	10.9 ± 2.7	6	3/3	25.0 ± 2.9	9.5 ± 1.4
C57B6 (wt)	8–15	3	2/1	29.4 ± 3.9	11.2 ± 0.5	5	4/1	26.9 ± 3.9	10.9 ± 1.0
C57B6 (wt)	18–24	6	5/1	31.7 ± 4.6	10.9 ± 1.3	6	5/1	31.7 ± 4.6	11.2 ± 0.9

The mice weights and injected doses in the microPET studies are expressed as means ± SD for each study group

*PET* positron emission tomography, *M* male, *F* female, *Inj.* injected, *wt* wild-type

### ^11^C-AZD2184 PET retention in APPswe mice

**Fig. 1:**
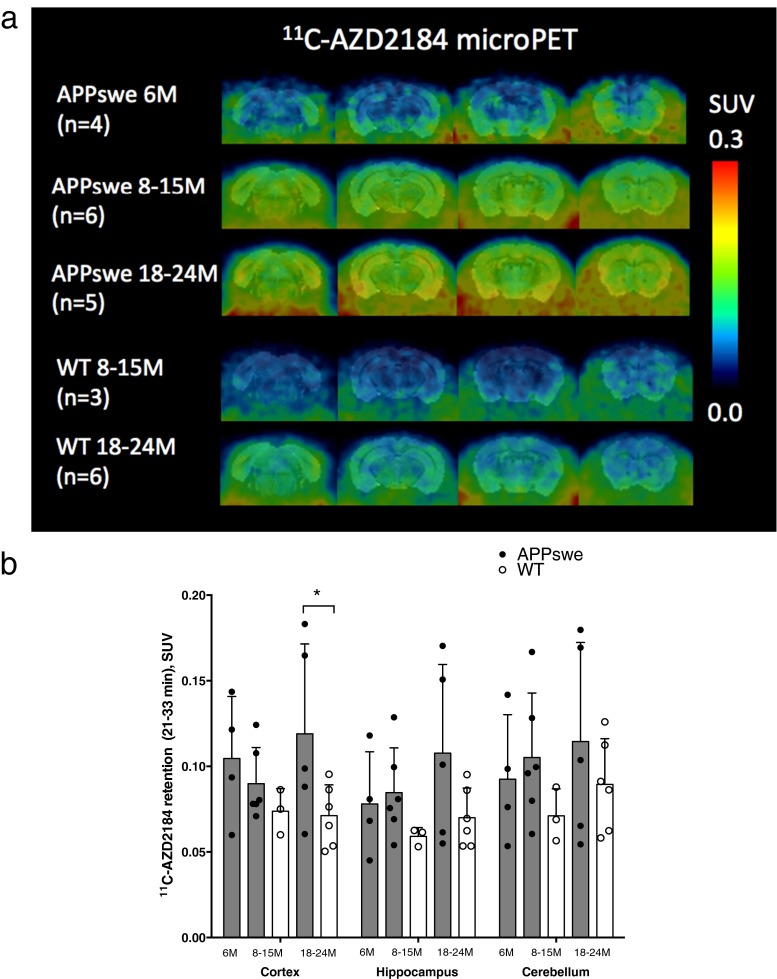
^11^C-AZD2184 microPET imaging in APPswe and wild-type (*WT*) mice. **a** ^11^C-AZD2184 microPET coronal images from three age groups of APPswe mice show greatest retention at 18–24 months (*n* = 5) and low retention in two groups of WT mice aged 8–15 months (*n* = 3) and 18–24 months (*n* = 6). Coronal images, depicted using a common scale from 0.0 to 0.3 SUV units, were obtained from averaged dynamic ^11^C-AZD2184 microPET scans (4–39 min post-injection for visualization purposes), subsequently combined into an average image for each mouse age group, and coregistered to a 3-D T2-weighted MRI mouse brain template using PMOD v3.3 software. Four coronal sections are shown for each group (*left to right*) at −5.2 mm, −3.2 mm, −1.2 mm and +0.8 mm from the bregma. **b** ^11^C-AZD2184 retention calculated from averaged dynamic microPET scans (21–33 min post-injection) expressed in SUV units for the cortices, hippocampi and cerebella from three groups of APPswe mice aged 6 months (*n* = 4), 8–15 months (*n* = 6) and 18–24 months (*n* = 5) and two groups of WT mice aged 8–15 months (*n* = 3) and 18–24 months (*n* = 6). Values in the columns are expressed as means ± SD. Significant differences between groups are indicated by *p < 0.05

Figure 1a displays average coronal images of ^11^C-AZD2184 PET scans in APPswe (aged 6, 8–15 and 18–24 months) and wt (aged 8–15 and 18–24 months) mice, coregistered to a 3-D T2-weighted MRI mouse brain template. ^11^C-AZD2184 retention expressed in SUV units showed increasing Aβ plaque deposition as a function of age in APPswe mice, with greater retention at 18–24 months. ^11^C-AZD2184 PET retention was significantly (67 %, *p* = 0.04) greater in 18- to 24-month-old APPswe mice cortices (mean SUV = 0.119 ± 0.053) than in age-matched wt mice cortices (mean SUV = 0.071 ± 0.018) (Fig. 1b). The comparison between ^11^C-AZD2184 retention in 18- to 24-month-old APPswe mice hippocampi (mean SUV = 0.108 ± 0.052) and in age-matched wt mice hippocampi (mean SUV = 0.070 ± 0.017) did not reach statistical significance (*p* = 0.12) (Fig. 1b). ^11^C-AZD2184 retention in a combined cortical and hippocampal ROI was significantly higher in 18- to 24-month-old APPswe compared to wt mice (*p* = 0.05). The ^11^C-AZD2184 retention in the cerebella of 18- to 24-month-old APPswe mice (mean SUV = 0.115 ± 0.058) did not significantly differ from that in age-matched wt mice (mean SUV = 0.089 ± 0.027) (*p* = 0.33). ^11^C-AZD2184 PET retention in the cortices, hippocampi and cerebella of 8- to 15-month-old APPswe mice did not differ significantly from that in 6-month-old APPswe or 8- to 15-month-old wt mice. Low ^11^C-AZD2184 brain retention was observed in all wt mice, irrespective of age (Fig. 1a, b).

### ^11^C-DED PET binding in APPswe and wt mice

**Fig. 2:**
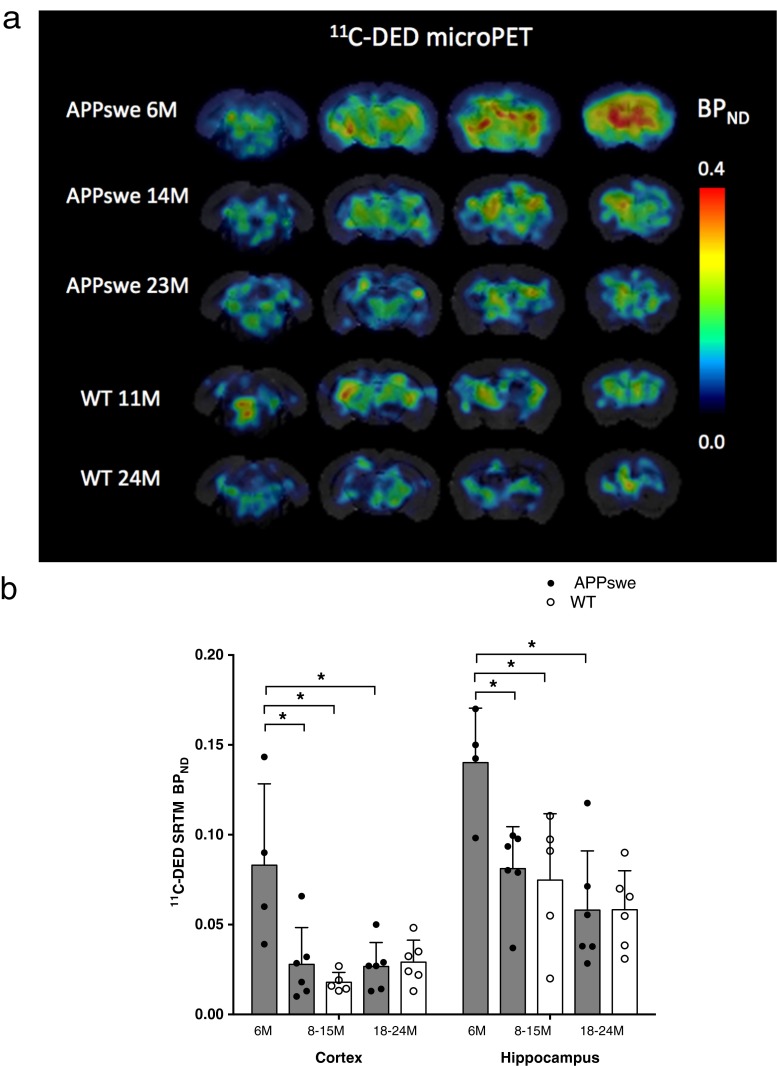
^11^C-DED microPET imaging in APPswe and wild-type (*WT*) mice. **a** ^11^C-DED microPET coronal images for APPswe mice show a different time course from that for WT mice, where ^11^C-DED binding was observed to decline with age in APPswe mice but not in WT mice. The coronal sections correspond to parametric BP_ND_ maps of ^11^C-DED PET smoothed by a 0.5-mm Gaussian filter and coregistered to a 3-D T2-weighted MRI mouse brain template using PMOD v3.3 software for representative APPswe compared to wt mice. All images are illustrated in a common scale from 0.0 to 0.4 units of BP_ND_. Four coronal sections are shown for each mouse (*left to right*) at −5.2 mm, −3.2 mm, −1.2 mm and +0.8 mm from the bregma. **b** ^11^C-DED binding in the cortex and hippocampus, expressed as BP_ND_, obtained from SRTM of ^11^C-DED using the cerebellum as a reference region, in three groups of APPswe mice aged 6 months (*n* = 4), 8–15 months (*n* = 6) and 18–24 months (*n* = 6) and two groups of WT mice aged 8–15 months (*n* = 5) and 18–24 months (*n* = 6). Values in the columns are expressed as means ± SD. Significant differences between groups are indicated by *p < 0.05

Figure 2a shows parametric BP_ND_ images of ^11^C-DED PET illustrated by coronal sections coregistered to a 3-D T2-weighted MRI mouse brain template for representative APPswe and wt mice. ^11^C-DED binding showed a different time course in APPswe compared to wt mice.

^11^C-DED binding was significantly greater in the cerebral cortices of 6-month-old APPswe mice (BP_ND_ = 0.083 ± 0.045) than in the 8- to 15-month-old cohort of wt mice (BP_ND_ = 0.018 ± 0.005; *p* = 0.014). ^11^C-DED binding was also significantly greater in the hippocampi of 6-month-old APPswe mice (BP_ND_ = 0.140 ± 0.030) than in the 8- to 15-month-old cohort of wt mice (BP_ND_ = 0.075 ± 0.037; *p* = 0.027) (Fig. 2b).

When comparing the three age groups of APPswe mice, ^11^C-DED binding was significantly higher in the cerebral cortices and hippocampi at 6 months compared to either 8- to 15- or 18- to 24-month-old APPswe mice. In the cerebral cortices, the ^11^C-DED binding at 6 months was significantly higher than at 8–15 months (BP_ND_ = 0.028 ± 0.020; *p* = 0.033) and than at 18–24 months (BP_ND_ = 0.027 ± 0.013; *p* = 0.019). In the hippocampi, the ^11^C-DED binding at 6 months was significantly higher than at 8–15 months (BP_ND_ = 0.081 ± 0.023; *p* = 0.019) and than at 18–24 months (BP_ND_ = 0.058 ± 0.033; *p* = 0.019).

^11^C-DED binding (BP_ND_) was observed to decline with age in APPswe mice in the cerebral cortices (Spearman’s *r*_S_ = −0.595; *p* = 0.015) and in the hippocampi (Spearman’s *r*_S_ = −0.707; *p* = 0.002), while BP_ND_ was not correlated with age in wt mice in either the cortical or hippocampal regions.

When ^11^C-DED binding was evaluated in semi-quantitative SUVR units, it was significantly greater in the cerebral cortices of 6-month-old APPswe mice (SUVR = 1.094 ± 0.063) than in the 8- to 15-month-old cohort of wt mice (SUVR = 1.036 ± 0.036; *p* = 0.05). There were no statistically significant differences in ^11^C-DED when expressed in SUVR units for any other comparison between groups of APPswe or wt mice in either the cerebral cortices or the hippocampi.

### ^3^H-AZD2184 and ^3^H-PIB in vitro binding in postmortem APPswe and wt mouse brain

**Fig. 3:**
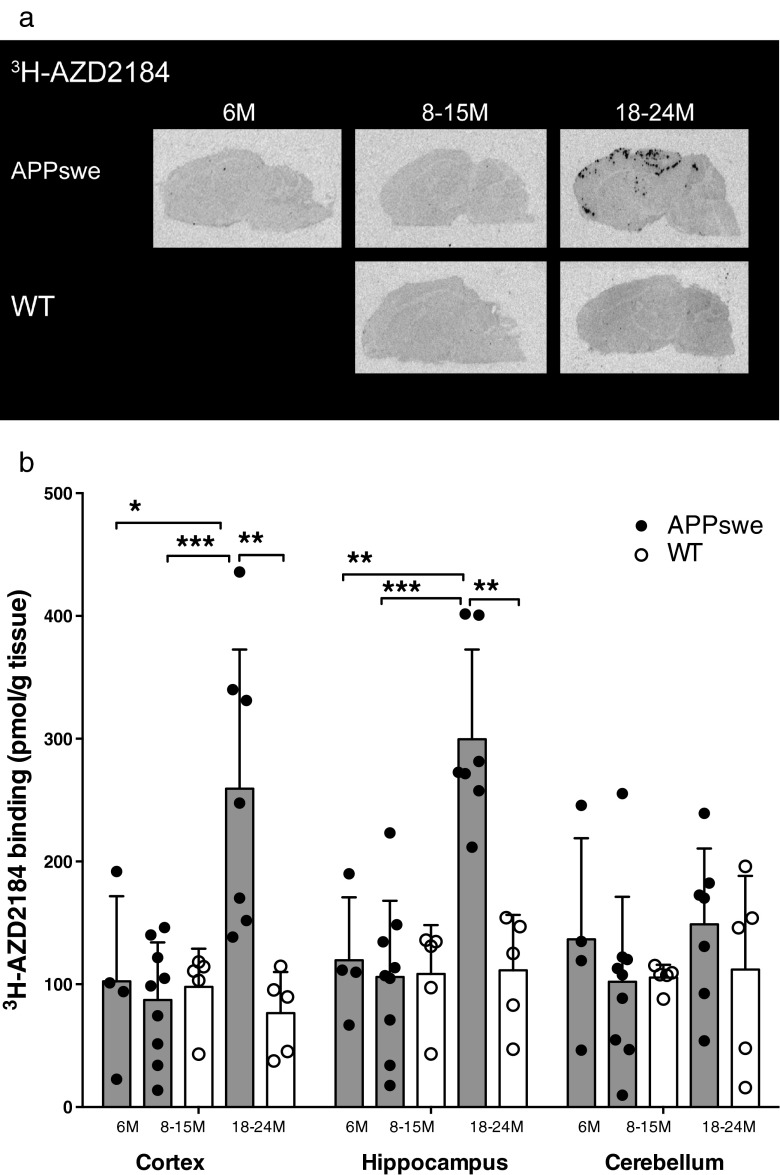
^3^H-AZD2184 in vitro binding in postmortem brains from APPswe and wild-type (*WT*) mice. **a** Representative ^3^H-AZD2184 (3 nM) autoradiograms in sagittal brain sections from APPswe (6, 11 and 23 months) and WT (11 and 18 months) mice. **b** ^3^H-AZD2184 binding (3 nM) in the cortices, hippocampi and cerebella of brains from APPswe mice aged 6 months (*n* = 4), 8–15 months (*n* = 9) and 18–24 months (*n* = 7) and WT mice aged 8–15 months (*n* = 5) and 18–24 months (*n* = 5). Values in the columns are expressed as means ± SD. Significant differences between groups are indicated by *p < 0.05, **p < 0.01 and ***p < 0.001

Saturation binding studies with ^3^H-AZD2184 demonstrated the presence of high-affinity binding sites (K_d_ 10.6 nM, B_max_ 530 pmol/g tissue) in the cerebral cortices of APPswe mice. The autoradiography binding profiles for ^3^H-AZD2184 in the brains of 6- to 24-month-old APPswe mice and 8- to 24-month-old wt mice are illustrated in Fig. 3a. More ^3^H-AZD2184 binding sites were detected in APPswe mice at 18–24 months than in age-matched wt mice; binding densities were higher in the hippocampi (299.6 ± 73.0 pmol/g tissue) and cerebral cortices (259.3 ± 113.3 pmol/g tissue) than in the cerebella (148.8 ± 61.7 pmol/g tissue) in these APPswe mice (Fig. 3a, b).

The number of ^3^H-AZD2184 binding sites in the cerebral cortices and hippocampi of 18- to 24-month-old APPswe mice was significantly greater than that in the 6-month-old (cortex *p* = 0.0424, hippocampus *p* = 0.0061) and 8- to 15-month-old (*p* = 0.0007, *p* = 0.0003) APPswe cohorts, respectively (Fig. 3b). ^3^H-AZD2184 binding levels were twofold higher in the cerebral cortices and the hippocampi of 18- to 24-month-old APPswe mice than in age-matched wt mice (cortex *p* = 0.0025, hippocampus *p* = 0.0025). There were no significant differences in ^3^H-AZD2184 binding densities in the cerebellum between APPswe and wt mice at any age (Fig. 3b).

**Fig. 4:**
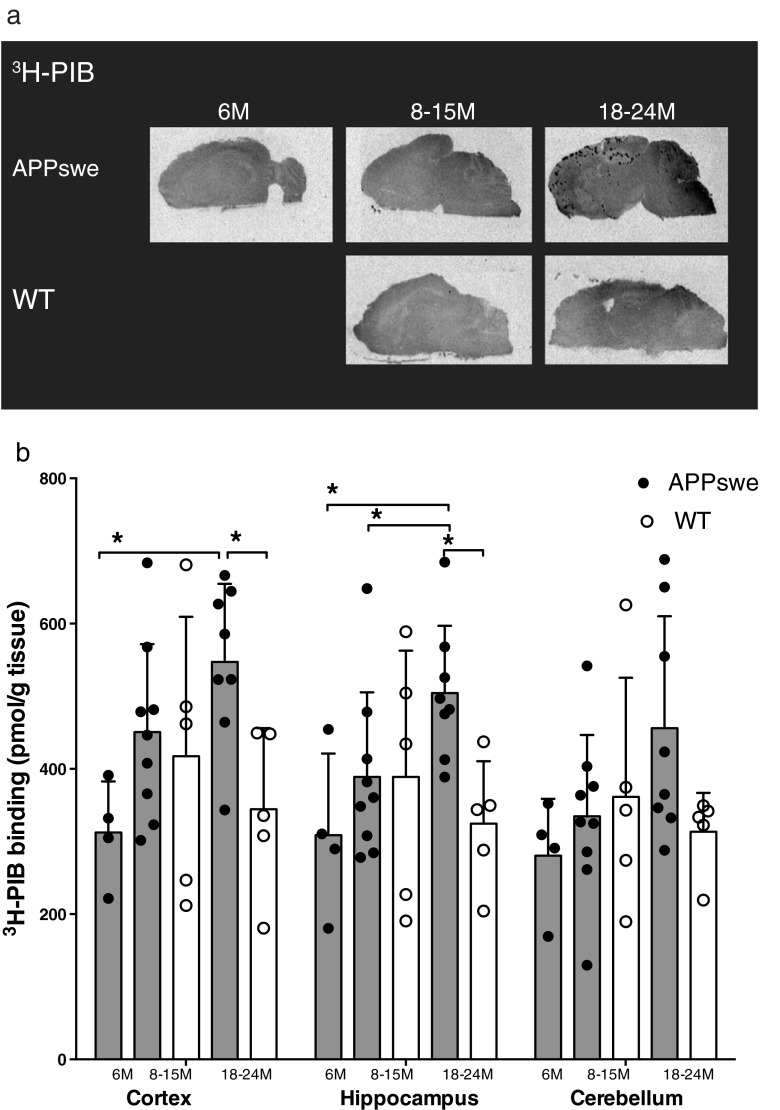
^3^H-PIB in vitro binding in postmortem brains from APPswe and wild-type (*WT*) mice. **a** Representative ^3^H-PIB (1.5 nM) autoradiograms in sagittal brain sections from APPswe (6, 11 and 23 months) and WT (11 and 18 months) mice. **b** ^3^H-PIB binding (1.5 nM) in the cortices, hippocampi and cerebella of brains from APPswe mice aged 6 months (*n* = 4), 8–15 months (*n* = 9) and 18–24 months (*n* = 8) and WT mice aged 8–15 months (*n* = 5) and 18–24 mths (*n* = 5). Values are expressed as means ± SD. Significant differences between groups are indicated by *p < 0.05

The amyloid ligand ^3^H-PIB was also used to quantify brain amyloid levels, and the profiles for ^3^H-PIB autoradiography binding in the brains of 6- to 24-month-old APPswe mice and 8- to 24-month-old wt mice are displayed in Fig. 4a. Higher levels of ^3^H-PIB binding were observed in 18- to 24-month-old APPswe mice, with higher binding densities in the cerebral cortices (547.2 ± 107.6 pmol/g tissue) and the hippocampi (504.3 ± 92.7 pmol/g tissue) and the lowest in the cerebella (456.1 ± 154.1 pmol/g tissue).

We found significantly higher levels of ^3^H-PIB binding in the cerebral cortices and hippocampi of 18- to 24-month-old APPswe mice (*p* = 0.0121, *p* = 0.0242) than in 6-month-old APPswe mice and higher ^3^H-PIB binding in the hippocampi of 18- to 24-month-old APPswe mice (*p* = 0.0311) than in 8- to 15-month-old APPswe mice (Fig. 4a, b). There were no significant differences in ^3^H-PIB binding levels in the cerebellum between 18- to 24-month-old APPswe mice and age-matched wt mice (Fig. 4b).

### ^3^H-L-deprenyl in vitro binding in postmortem APPswe and wt mouse brain

^3^H-L-deprenyl saturation binding revealed the presence of high-affinity binding sites in the cortices of 6-month-old (K_d_ 9.5 nM, B_max_ 201 pmol/g tissue) and 20-month-old (K_d_ 16.0 nM, B_max_ 238 pmol/g tissue) APPswe mice.

**Fig. 5:**
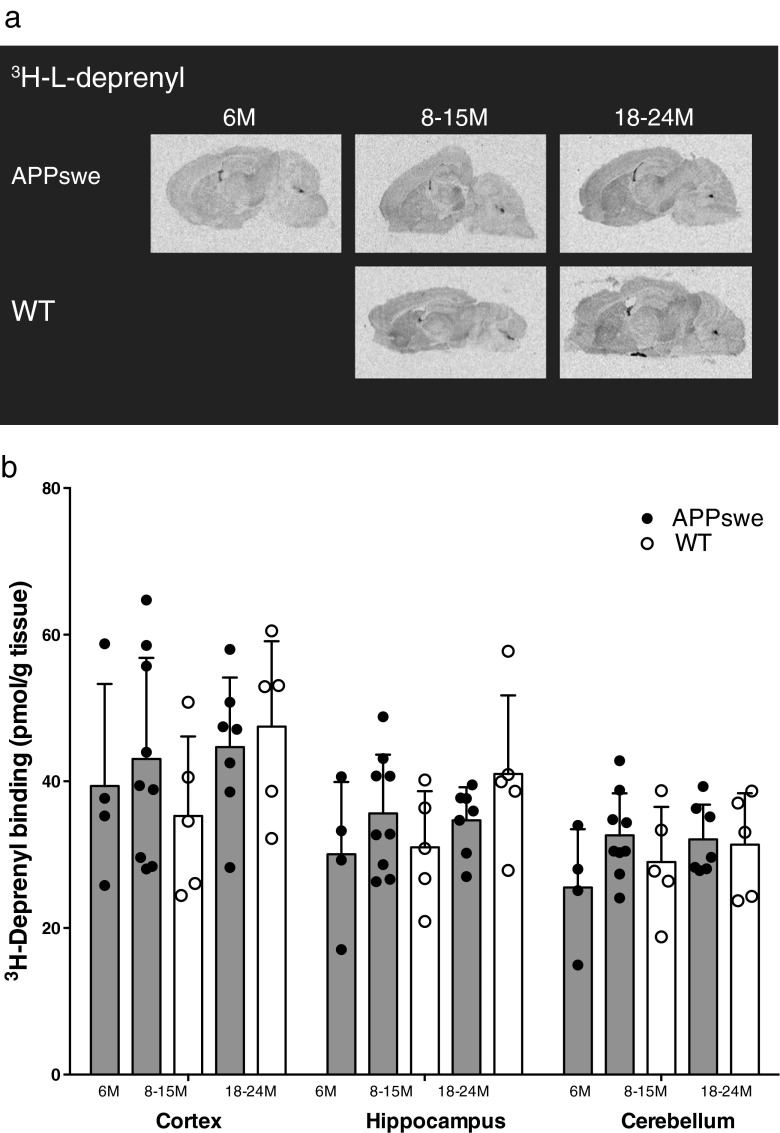
^3^H-L-deprenyl in vitro binding in postmortem APPswe and wild-type (*WT*) mouse brain. **a** Representative ^3^H-L-deprenyl (10 nM) autoradiograms in sagittal brain sections from APPswe (6, 11 and 23 months) and WT mice (11 and 18 months). **b** ^3^H-L-deprenyl (10 nM) in the cortices, hippocampi and cerebella of brains from APPswe mice aged 6 months (*n* = 4), 8–15 months (*n* = 9) and 18–24 months (*n* = 7) and WT mice aged 8–15 months (*n* = 5) and 18–24 months (*n* = 5). Values are expressed as means ± SD

The autoradiography binding profiles for ^3^H-L-deprenyl in sagittal brain sections from 6- to 24-month-old APPswe and 8- to 24-month-old wt mice are shown in Fig. 5a. ^3^H-L-deprenyl demonstrated similar binding levels in both the cortex and hippocampus across the different age groups of APPswe mice that were comparable to the levels in wt mice (Fig. 5b).

### Distribution of Aβ_42_ deposits and GFAP-immunoreactive astrocytes in APPswe mice brains

**Fig. 6:**
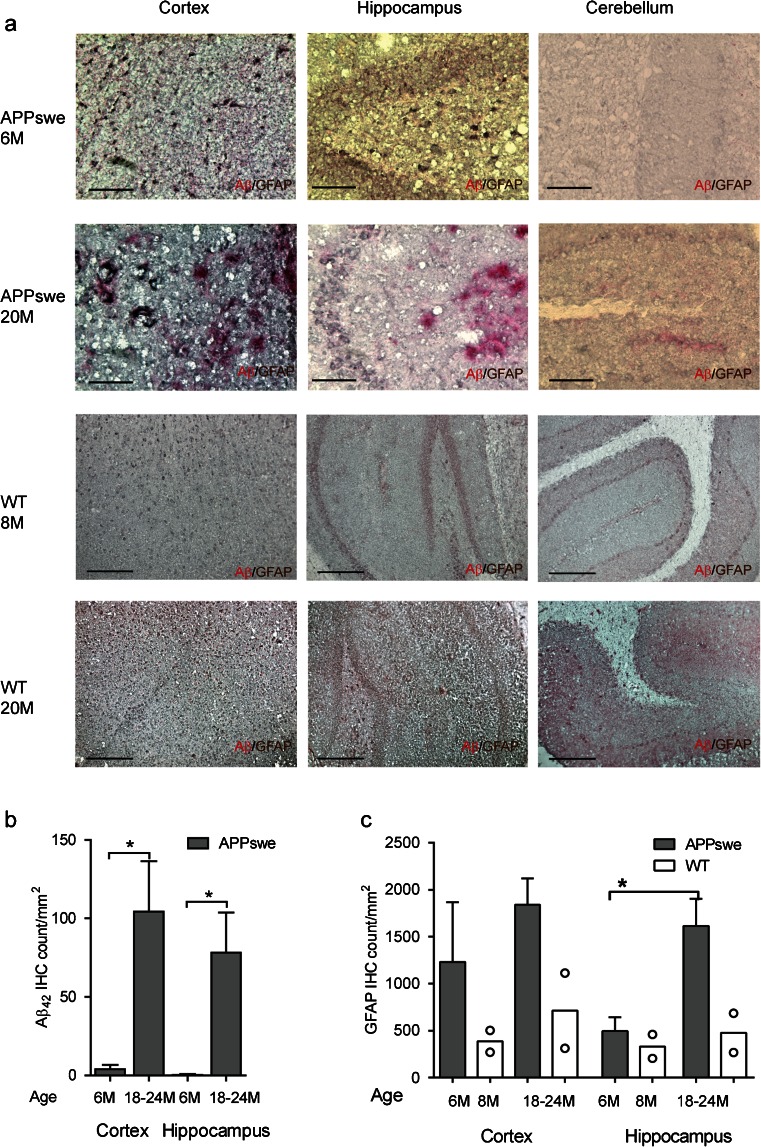
Immunohistochemical staining in APPswe mice. **a** Representative immunohistochemical staining of Aβ aggregates and GFAP^+^ reactive astrocytes in the cortex, hippocampus (dentate gyrus) and cerebellum of one 6-month-old and one 20-month-old APPswe mouse (×20), *scale bars* = 0.25 mm, and one 8-month-old and one 20-month-old wild-type (*WT*) mouse (×10), *scale bars* = 0.5 mm; GFAP^+^ astrocytes (*brown*) surround Aβ_42_ positive plaques (*red*). **b** Higher levels of Aβ_42_ in the cortices and hippocampi of 18- to 24-month-old APPswe mice (*n* = 4) than in those of 6-month-old APPswe mice (*n* = 4). No Aβ_42_ aggregates were observed in the cortex and hippocampus of the 8-month-old and 20-month-old WT mice. **c** Higher levels of GFAP in the hippocampi (dentate gyri) of 18- to 24-month-old APPswe mice (*n* = 4) than in those of 6-month-old APPswe mice (*n* = 4). GFAP levels of age-matched WT mice are included for comparison. Values are expressed as means ± SEM. Significant differences between groups are indicated by *p < 0.05

Aβ_42_ immunostaining showed more Aβ_42_ deposits in the cortices and hippocampi of 18- to 24-month-old APPswe mice than in 6-month-old APPswe mice (Fig. 6a, b). Scarce diffuse Aβ_42_ deposits were observed in the cortices and hippocampi of 6-month-old APPswe mice, whereas increased numbers of both diffuse and cored Aβ_42_ plaque deposits were detected in 18- to 24-month-old APPswe mice (Fig. 6a, b). Diffuse Aβ_42_ deposits were also detected in the cerebella of APPswe mice at 18–24 months of age, but these were morphologically different from those observed in the cortices and hippocampi, and were mainly distributed along blood vessels and meninges (Fig. 6a). No Aβ_42_ aggregates were observed in the cortex and hippocampus of the 8-month-old and 20-month-old wt mice (Fig 6a).

GFAP^+^ astrocytes were significantly more abundant in the hippocampi of APPswe mice at 18–24 months than at 6 months, while the difference was not statistically significant in the cortex (Fig. 6c). Age-dependent changes in the morphology and phenotype of GFAP^+^ reactive astrocytes were also observed. In the cortices and hippocampi of 6-month-old APPswe mice, in the absence of extracellular Aβ_42_ plaques, the majority of GFAP^+^ reactive astrocytes were atrophic and a large proportion of these contained cytoplasmic Aβ_42_. However, in the cortices and hippocampi of 18- to 24-month-old APPswe mice, both atrophic and hypertrophic GFAP^+^ reactive astrocytes were detected, with only a few of these containing cytoplasmic Aβ_42_. The hypertrophic astrocytes in the cortices and hippocampi of 18- to 24-month-old APPswe mice accounted for about 10 % of the total reactive astrocytes and were found surrounding Aβ_42_-stained amyloid plaques, while the atrophic astrocytes were observed distant from the Aβ_42_ amyloid plaques. GFAP^+^ reactive astrocytes were lower in the cortex and hippocampus of wt mice compared to APPswe mice at both 8 months of age and 18–24 months of age (Fig 6a, c).

## Discussion

The use of multitracer PET imaging in an AD transgenic mouse model to investigate the evolution of and relationships between astrocytosis and amyloid plaque deposition in early AD has, to our knowledge, not been described before, as indicated also in a recent review [27]. Previous amyloid PET studies in AD transgenic mice have reported various findings [27]. For example, retention of ^11^C-PIB was no greater in the brains of 12-month-old APP/PS1 mice than in wt mice in one study [28], while subsequent studies reported increased ^11^C-PIB retention from 17 to 18 months in APP23 mice [29, 30], but not in 22-month-old APPswe mice [30, 31]. Other similar studies in APP/PS1 mice showed increased retention at 9 months [32] and no difference at 15–22 months compared to age-matched wt mice [30]. Increased PET retention of ^18^F-florbetaben in 16-month-old APPswe mice [33] and ^18^F-florbetapir in 5-month-old APP/PS1 mice [34] has been reported.

In this study, we used PET imaging to detect increased ^11^C-AZD2184 retention in older APPswe mice. These in vivo studies showed a significant increase in retention in the cortices of 18- to 24-month-old APPswe mice versus age-matched wt mice, and a similar increase in both the cortex and the hippocampus was observed postmortem with ^3^H-AZD2184 and ^3^H-PIB in vitro autoradiography. Age-related increases in ^3^H-AZD2184 binding densities, as well as with ^3^H-PIB, and Aβ_42_ immunopositive deposits were also observed in the cortices and hippocampi of APPswe mice, in agreement with previously published findings of Aβ deposition in postmortem immunohistochemical studies in this model [35].

The gene expression and cellular morphology of reactive astrocytes are highly heterogeneous across different brain regions in both healthy and neurodegenerative- diseased brains [36, 37]. Studies in AD transgenic mice have reported widespread astroglial atrophy in the brain in the earlier stages of AD prior to Aβ plaque deposition [38–40], but have also indicated the presence of hypertrophic astrocytes surrounding amyloid plaques [41]. The Aβ aggregates in the brain increase the number of reactive astrocytes and/or phenotypic changes and upregulate GFAP expression [42, 43].

Our ^11^C-DED PET imaging findings demonstrated different time courses for astrocytosis in APPswe and wt mice; ^11^C-DED binding significantly decreased with age in APPswe mice in cortex and hippocampus, while it was not correlated with age in wt mice. ^11^C-DED binding was also significantly higher in the cortices and hippocampi of 6-month-old APPswe mice compared to other age groups of APPswe and compared to 8- to 15-month-old wt mice. These findings and the observed absence of Aβ plaques in 6-month-old APPswe mice indicate that astrocytosis occurs prior to Aβ plaque deposition.

Previous studies have measured reactive astrocytes using ^3^H-L-deprenyl in vitro in AD postmortem brain tissue [26, 44, 45] and ^11^C-DED in vivo [18, 46]. Interestingly, early ^11^C-DED PET binding was observed in prodromal AD patients [18], consistent with our results in APPswe mice. Astrocytosis as measured by ^11^C-DED PET was also observed to be negatively correlated with grey matter density in the parahippocampus at early prodromal AD stages [47]. Early ^11^C-DED PET binding has also been reported in presymptomatic carriers of autosomal dominant AD mutations several decades before onset of symptoms and earlier than Aβ deposition [48]. Therefore, our findings of early ^11^C-DED PET binding in APPswe mice before Aβ deposition are consistent with reported in vivo PET findings in humans, highlighting the translational aspects of this study.

In contrast to the in vivo ^11^C-DED PET binding results, there were no significant differences between APPswe and wt mice in the in vitro ^3^H-L-deprenyl autoradiography results. Possible explanations for this include either a reduction in monoamine oxidase B (MAOB) enzyme activity when measured in vitro compared to in vivo [46] or technical limitations due to a reduced in vitro enzyme activity in transgenic APPswe mice compared to humans.

The level of GFAP upregulation in astrocytes has been reported to be dependent on both the brain region and the context, which could in part underlie the heterogeneity of functions mediated by astrocytes in the central nervous system, evidenced by their diverse neuroprotective or neurotoxic functions across different stages in AD [49]. Our immunostaining results in APPswe mice revealed more GFAP reactive astrocytes in the cortices and hippocampi of 18- to 24-month-old than of 6-month-old APPswe mice, although the age-dependent increase was significant only in the hippocampus. There is increasing evidence that GFAP is not expressed uniformly by all astrocytes [50] and that different GFAP isoforms develop in response to plaque-related gliosis, as shown in APP/PS1 and 3xTg AD transgenic mice [51]. It has been reported [52] that about 80 % of the astrocytes in the hippocampus express GFAP, compared to only 15–20 % of those in the cortex, which could at least partially explain our immunohistochemical results showing a significant increase in GFAP with age in the hippocampus. Other studies in AD transgenic mice have reported GFAP expression prior to Aβ plaque deposition [53] and also as an age-dependent process that is correlated with oligomeric Aβ but not with plaque burden [54]. GFAP expression in astrocytes has also been reported as a late event related to plaque formation and maturation, and as a neuroprotective event that limits Aβ plaque growth [55].

In our present study, in contrast to the early increase in ^11^C-DED binding in the APPswe mouse brain cortex, GFAP immunoreactivity was predominantly a late event. There was a significant age-dependent increase in the number of GFAP^+^ reactive astrocytes in the hippocampus, possibly indicating the presence of distinct subpopulations of astrocytes and/or different stages of reactive astrocytosis. The different time course of increases in ^11^C-DED binding and GFAP upregulation observed in APPswe mice is consistent with findings in human AD. Quantitative autoradiography studies in postmortem AD brains have shown a strong regional correlation between the number of GFAP^+^ reactive astrocytes and the extent of in vivo ^11^C-PIB and in vitro ^3^H-PIB binding, but there appears to be no regional correlation between postmortem ^3^H-L-deprenyl and in vivo ^11^C-PIB binding [45]. The regional and laminar distribution patterns of ^3^H-L-deprenyl reactive astrocytes also differed from those of fibrillar Aβ in AD autopsy brains [26]. Thus, our observation of early ^11^C-DED binding in the cortices of APPswe mice might reflect the presence of a subset of activated astrocytes that is functionally different from those measured by GFAP at later stages.

The small, soluble Aβ forms that have been observed in APPswe mice from birth [56, 20] could influence astrocyte function. For example, Aβ_25–35_ peptides caused overexpression of MAOB in cultured rat astrocytes [57]. Further, MAOB overexpression in astrocytes led to production of proinflammatory molecules contributing to exacerbated neuroinflammation and Aβ plaque formation at later stages [58]. Our observation of early elevated ^11^C-DED binding might thus reflect a reaction of astrocytes to these small Aβ forms, with potential beneficial and/or neurotoxic consequences. Activated astrocytes appear to play a role in the clearance of Aβ [59, 60]. Whether the reactive astrocytes that were observed early in the development of AD, as measured by elevated ^11^C-DED binding, have a phagocytic function requires further investigation.

One limitation of this study was the limited spatial resolution of small animal PET imaging relative to the mouse brain regions selected for quantification, which contained some thin shapes being susceptible to partial volume effects. This might account for the lower sensitivity of our in vivo versus in vitro images. Inevitably, transgenic AD mice are an inherently limited model for human disease and APPswe mice might phenotypically reflect only some aspects of AD [19]. Astrocytosis was elevated at 6 months and it subsequently declined with age in APPswe mice, consistent with findings of increased astrocytosis in the prodromal phase of AD followed by decreases in later stages in humans [18]. The cross-sectional design of this study allowed a parallel in vivo/in vitro comparison of the regional and temporal distributions of Aβ deposition and astrocytosis in the same APPswe and wt mice at a given age interval. Further multitracer longitudinal PET imaging studies in AD transgenic animal models could provide additional insights into the temporal evolution of neuropathological changes during AD progression and could be useful for testing new therapies targeting astrocytes, especially at the earliest stages.

In conclusion, we provide in vivo evidence that astrocytosis occurs early in AD and precedes Aβ plaque deposition. The increasing recognition of heterogeneous and context-dependent astrocytosis in the progression of AD indicates that more research is needed to elucidate the functions of the different astrocyte populations in the brain at different stages of the disease.

## Electronic supplementary material

ESM 1 (PDF 110 kb)
