# Supplementary material for: Astrocytosis precedes amyloid plaque deposition in Alzheimer APPswe transgenic mouse brain: a correlative positron emission tomography and in vitro imaging study
Source: Eur J Nucl Med Mol Imaging. 2015 Apr 17;42(7):1119–32. doi: 10.1007/s00259-015-3047-0 (PMC4424277; doi:10.1007/s00259-015-3047-0)
Supplement: Supplementary file 1 — (PDF 110 kb) [file 259_2015_3047_MOESM1_ESM.pdf]

Supplementary Material for

Astrocytosis precedes amyloid plaque deposition in Alzheimer APPswe transgenic mouse brain: a correlative positron emission tomography and *in vitro* imaging study

European Journal of Nuclear Medicine and Molecular Imaging

Elena Rodriguez-Vieitez*, Ruiqing Ni*, Balázs Gulyás, Miklós Tóth, Jenny Häggkvist, Christer Halldin, Larysa Voytenko, Amelia Marutle, Agneta Nordberg

*Contributed equally to this work.

Correspondence to Professor Agneta Nordberg
Karolinska Institutet, Division of Translational Alzheimer Neurobiology, Karolinska University Hospital Huddinge, Novum 5th Floor, Blickagången 6,
S-141 57 Stockholm, Sweden.
Telephone: +46 8 585 85467
Fax: +46 8 585 85470
E-mail: Agneta.K.Nordberg@ki.se

This PDF file includes:

Supplementary Fig. S1 Representative time-activity curves of ^{11}C-AZD2184 and ^{11}C-deuterium-L-deprenyl (^{11}C-DED) radiotracers in APPswe and wild-type mice

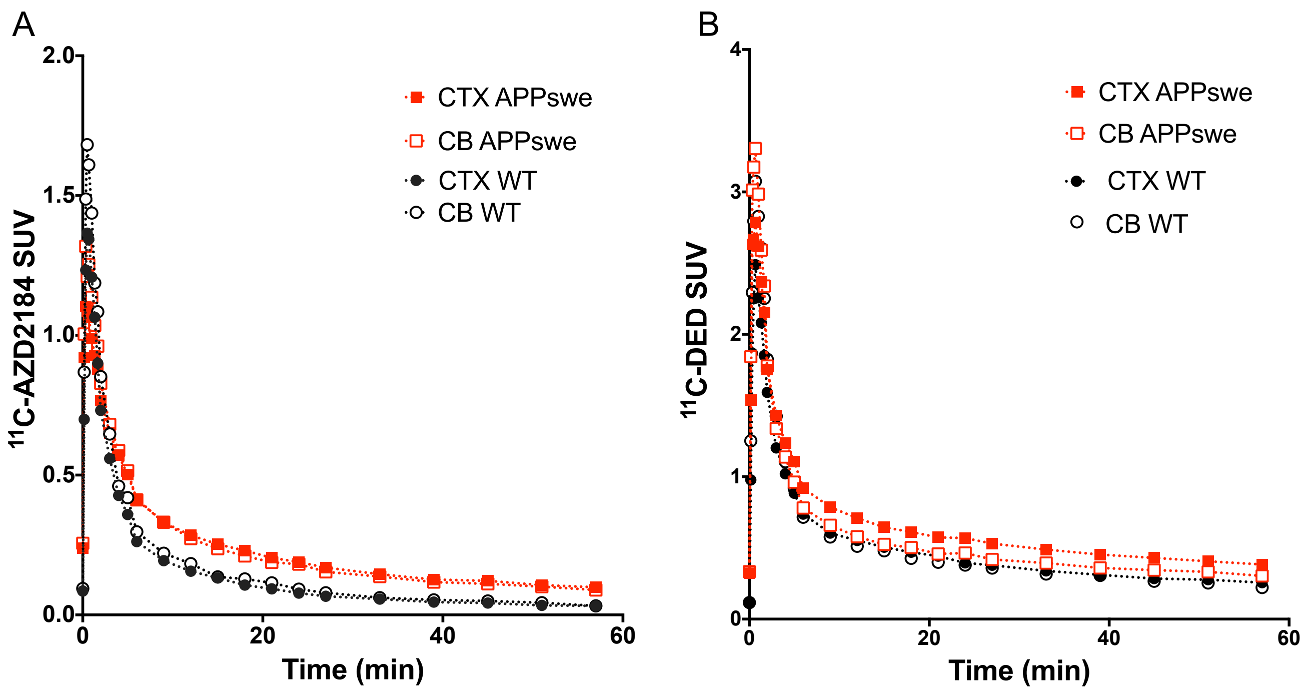

Supplementary Fig. S1 Representative time-activity curves of ^{11}C-AZD2184 and ^{11}C-deuterium-L-deprenyl (^{11}C-DED) radiotracers in APPswe and wild-type mice. **A)** Time-activity curves of ^{11}C-AZD2184 in 20-month APPswe and age-matched wild-type mice. **B)** Time-activity curves of ^{11}C-DED in 6-month APPswe and 8-month wild-type mice. CTX = cortex; CB = cerebellum; WT = wild-type; SUV = standard uptake value
